# Metformin Derivative HL156A Reverses Multidrug Resistance by Inhibiting HOXC6/ERK1/2 Signaling in Multidrug-Resistant Human Cancer Cells

**DOI:** 10.3390/ph13090218

**Published:** 2020-08-28

**Authors:** Yun Soo Jeong, Thuy Giang Lam, Seho Jeong, Sang-Gun Ahn

**Affiliations:** Department of Pathology, School of Dentistry, Chosun University, Gwangju 61452, Korea; yeonsoo1660@gmail.com (Y.S.J.); lamgiang1612@gmail.com (T.G.L.); hoho804kr@gmail.com (S.J.)

**Keywords:** metformin, HL156A, multidrug resistance, HOXC6, angiogenesis

## Abstract

Multidrug resistance is a significant clinical crisis in cancer treatment and has been linked to the cellular expression of multidrug efflux transporters. The aim of this study was to examine the effects and mechanisms of the metformin derivative HL156A on human multidrug resistance (MDR) cancer cells. Here, HL156A significantly suppressed cell growth and colony formation through G2/M phase cell cycle arrest in MDR cancer cells. HL156A also reduced the wound closure rate and cell migration and induced caspase-3-dependent apoptosis. We found that HL156A inhibited the expression of MDR1 by inhibiting the HOXC6-mediated ERK1/2 signaling pathway and increased the sensitivity to paclitaxel or doxorubicin in MDR cells. Furthermore, HL156A significantly inhibited angiogenesis in a chicken chorioallantoic membrane (CAM) assay. These results suggest the potential of the metformin derivative HL156A as a candidate therapeutic modality for the treatment of human multidrug-resistant cancers.

## 1. Introduction

Chemotherapy treatment is often associated with multidrug resistance (MDR), resulting in unsatisfactory treatment outcomes such as intractable tumors or cancer recurrence [1,2]. Therefore, multidrug resistance is an obvious target for cancer research in an attempt to improve clinical outcomes. Generally, multidrug resistance to anticancer drugs can be categorized into two classes: impaired anticancer drug delivery due to poor absorption, increased drug metabolism or increased secretion; and reduced drug sensitivity due to genetic and epigenetic alterations. MDR is the phenomenon in which simultaneous cross-resistance to structurally and mechanistically distinctive drugs is stimulated after selective resistance to a single drug. Importantly, more than one mechanism of multidrug resistance can be present in any population of cancer cells due to their genetically heterogeneous nature. Several studies have proposed cellular mechanisms of MDR, including increased efflux, reduced influx, activation of coordinately regulated detoxifying systems, DNA repair activation and defective apoptotic pathways [3,4,5,6,7]. The expression of ATP-dependent efflux pumps (ATP-binding cassette (ABC) transporters) is considered to be one of the most commonly reported mechanisms of MDR. Among the ABC transporters, P-glycoprotein (P-gp or ABCB1) is a 170 kDa membrane protein encoded by the MDR-1 gene that has broad cross-resistance specificity for anticancer drugs, including doxorubicin, cisplatin, and Taxol (paclitaxel) [2,3,8]. P-gp prevents the sufficient accumulation of intracellular anticancer drugs by ATP-dependent efflux and helps cancer cells avoid cytotoxic or apoptotic effects [8,9,10]. Due to its key involvement in MDR, it is exceptionally essential to generate compounds or strategies to overcome the actions of P-gp, to restore chemotherapy sensitivity and to design compounds with better pharmacokinetic profiles for future anticancer drugs. The MDR-1 gene is the downstream target for well-known factors such as p53, YB-1 and NF-ĸB [11,12]. Recent studies have shown that PI3K-AKTsignaling and Wnt/β-catenin signaling promote the expression of MDR-1 mRNA by inducing the transcription of the MDR-1 gene, while the activation of the MAPK/p38, ERK and JNK/c-Jun/AP-1 pathways regulates MDR-1 expression through both transcriptional and posttranscriptional modifications [13,14].

Metformin is a biguanide-based drug that is most commonly described in patients with type 2 diabetes. Metformin has been extensively studied for its anticancer effects, and research on metformin in diabetic patients reports that it can reduce the incidence of cancer [15]. Metformin is well known as an AMP-activated protein kinase (AMPK) activator that regulates glucose and lipid metabolism, inflammation regulation, cell proliferation and cell death through interaction with a variety of other factors, such as AKT and mTOR [16,17]. Several preclinical and clinical studies have shown the beneficial effects of metformin in chemoresistance. In a recent study, metformin prevented chemoresistance in patients with breast cancer who were administered doxorubicin-based treatments [18,19]. Additionally, metformin enhanced the inhibition of MDR in hepatocellular carcinoma via the downregulation of P-gp and MRP-1 expression [20]. However, the mechanisms involved in the capacity of antidiabetic drugs to sensitize MDR cells have yet to be clarified.

Regardless of its anticancer properties, metformin does not enter cells efficiently due to its hydrophilicity [21]. HL156A is a newly synthesized derivative of metformin that has higher bioavailability and elicits more potent AMPK activation than metformin [22,23]. It has been reported that HL156A elicits not only antifibrotic effects in peritoneal and liver fibrosis but also protective effects against LPS-induced inflammation [23,24,25]. In addition, HL156A has been shown to inhibit epithelial–mesenchymal transition (EMT) as well as Smad3-dependent pathways activated by high glucose conditions [25]. Interestingly, the combination of HL156A and temozolomide in glioblastoma inhibited the proliferation of cancer stem cells and increased survival in animal models [23]. Moreover, our recent study confirmed that HL156A effectively inhibited the progression of oral cancer by inhibiting the IGF/AKT/mTOR signaling pathway [26]. HL156A also exhibited antioxidant effects in an accelerated aging mouse model by inducing glutathione metabolism and antioxidant pathways [27].

In this study, we observed that HL156A had a greater effect on the proliferative and metastatic potential of paclitaxel-resistant head and neck squamous cell carcinoma FaDu/PTX cells and adriamycin-resistant breast cancer MCF7/ADR cells than metformin. Collectively, we indicated that HL156A has the potential to be considered as a therapeutic agent for MDR cancer treatment.

## 2. Results

### 2.1. Effects of HL156A on MDR Cell Proliferation

**Figure 1:**
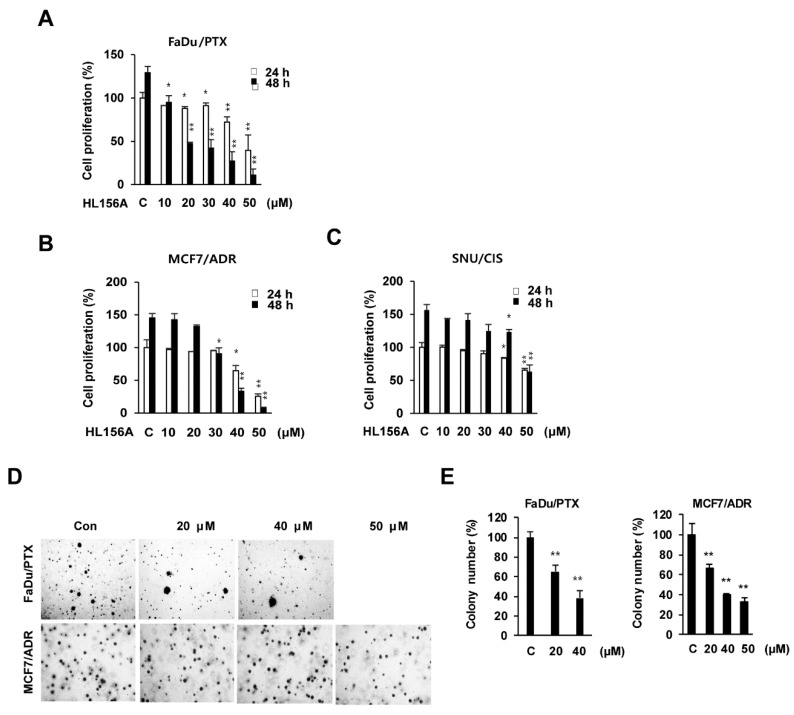
Effects of HL156A on cell proliferation in human multidrug-resistant cancer cells. (**A**–**C**) FaDu/PTX, MCF7/ADR, and SNU601/CIS cells were treated with HL156A (10 to 50 μM) for 24 h or 48 h. Cell proliferation was assessed using the MTT assay. The data are expressed as the mean ± SD of the results from three separate experiments (* *p* < 0.05 and ** *p* < 0.01). Data were compared by ANOVA with Bonferroni’s multiple comparisons test. (**D**) Evaluation of the colony formation of HL156A-treated cells. Colony formation was assessed 14 days after HL156A treatment at the indicated concentrations, and cells were stained with crystal violet at the end of the experiment. Images were taken with an inverted microscope at ×40 magnification. (**E**) The number of colonies in each agar plate was graphed. Values are presented as the mean ± SD. * *p* < 0.05 and ** *p* < 0.01. Data were compared by ANOVA with Bonferroni’s multiple comparisons test.

To investigate the effect of HL156A on the proliferation of MDR cells, MTT assays were performed in FaDu/PTX, MCF7/ADR, and SNU601/CIS cells. HL156A significantly reduced the cell proliferation of these cells in a concentration-dependent or time-dependent manner (Figure 1A–C). The IC_50_ values of HL156A were around 40 µM/mL in FaDu/PTX and MCF7/ADR at 24 h. However, SNU601/CIS cells were 84.9 µM/mL. In addition, the IC_50_ values of HL156A were 15, 31.7, and 55.4 µM/mL for FaDu/PTX, MCF7/ADR, and SNU601/CIS cells at 48 hr, respectively. After treatment with 40 μM HL156A, FaDu/PTX and MCF7/ADR cell proliferation was decreased by 72.8% and 67% at 48 h compared to the control, respectively. In SNU601/CIS cells, this inhibition was induced by higher concentrations of HL156A (≥50 μM). Therefore, Fadu/PTX and MCF7/ADR cell lines with high sensitivity to HL156A were selected and used in further experiments. We also performed a BrdU incorporation assay to assess cell growth. HL156A treatment significantly decreased cell growth in FaDu/PTX and MCF7/ADR cells, suggesting that HL156A additively decreased DNA synthesis in both cells (Figure S1). We clearly confirmed the inhibition of cell proliferation by HL156A in the BrdU assay and the MTT assay.

In addition, we examined the effects of metformin on parental cells (FaDu, MCF7 and SNU601) and their MDR counterparts. In FaDu/PTX and MCF7/ADR, metformin decreased cell proliferation at high concentrations (≥50 mM). Of note, in SNU601/CIS cells, metformin did not affect the inhibition of cell proliferation (Figure S2). Interestingly, HL156A exhibited better inhibitory effects than metformin at lower concentrations in parental cells (Figure S3). Our results showed that HL156A inhibited MDR cell proliferation more potently than metformin.

A soft agar colony formation assay also confirmed the inhibitory effects of HL156A on cell growth over 14 days (Figure 1D). Cell clonogenicity was inhibited by 62% and 55% in FaDu/PTX and MCF7/ADR cells, respectively, following treatment with 40 μM HL156A, as compared to the control group (Figure 1E).

### 2.2. HL156A Induces G2/M Cell Cycle Arrest and Apoptosis

**Figure 2:**
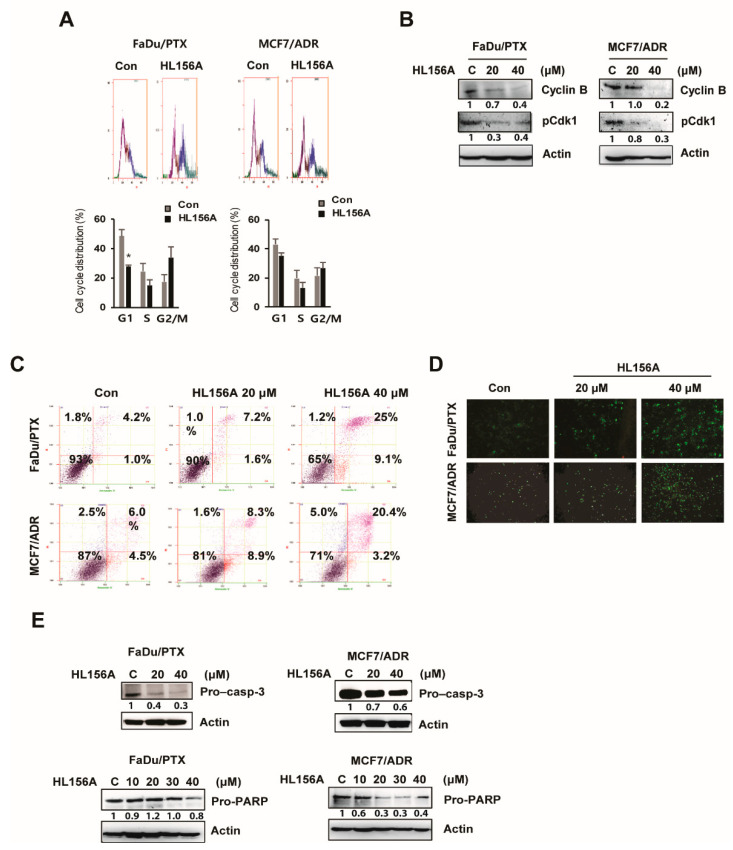
HL156A induces G2/M phase cell cycle arrest and apoptosis. (**A**) Cells were treated with HL156A (40 μM) for 24 h and then subjected to flow cytometry to measure cell cycle distribution. The percentage of cells in each cell cycle phase was graphed. Student’s t-tests were used to determine the significance. (**B**) Immunoblotting of cell cycle-related proteins. FaDu/PTX and MCF7/ADR cells were treated with 20 or 40 μM HL156A for 24 h. Lysates of the above cells were subjected to Western blotting with phospho-CDK1 and cyclin B antibodies. β-actin served as an internal control. (**C**,**D**) Apoptotic cells were assessed by flow cytometric analysis and fluorescence microscopy after annexin V/PI double staining. (**E**) The effect of HL156A on the activation of caspase 3 and PARP. The cells were treated with HL156A for 24 hr, and procaspase-3 and pro-PARP were measured by Western blotting in FaDu/PTX and MCF7/ADR cells.

To examine whether HL156A affects cell cycle progression, we performed flow cytometry analysis of HL156A-treated MDR cells. As shown in Figure 2A, HL156A treatment resulted in a G2/M population increase in both FaDu/PTX and MCF7/ADR cells, while the G1 and S phase population decreased. Furthermore, the levels of phospho-CDK1 and cyclin B, major regulators of the G2/M phase, were decreased in HL156A-treated cells in a concentration-dependent manner (Figure 2B).

To investigate whether HL156A induces cell death, an annexin V-FITC/PI double staining assay was performed, and cell death was quantified using flow cytometry. As shown in Figure 2C, a decrease in live cell number was observed after treatment with HL156A. In FaDu/PTX cells treated with 20 μM HL156A, 90% viable cells and 1.6% apoptotic cells were observed, while a higher concentration of HL156A (40 μM) showed a more apparent effect with 65% viable cells and 9.1% apoptotic cells. Similarly, there was a noticeable increase in the proportion of apoptotic cells (8.9% with 20 μM) in MCF7/ADR cells compared to the untreated control. As expected, many annexin V-FITC-positive cells were observed in HL156A-treated cells compared to the control using fluorescence microscopy (Figure 2D). However, SNU/CIS cells were not significantly affected by the same concentration of HL156A (Figure S4).

Consistent with this observation, Western blot analysis revealed that levels of the inactive forms of procaspase-3 and poly (ADP-ribose) polymerase (PARP) were reduced in a concentration-dependent manner in cells treated with HL156A compared to control cells (Figure 2E).

### 2.3. HL156A Inhibits the Migration and Invasion Ability of MDR Cells

**Figure 3:**
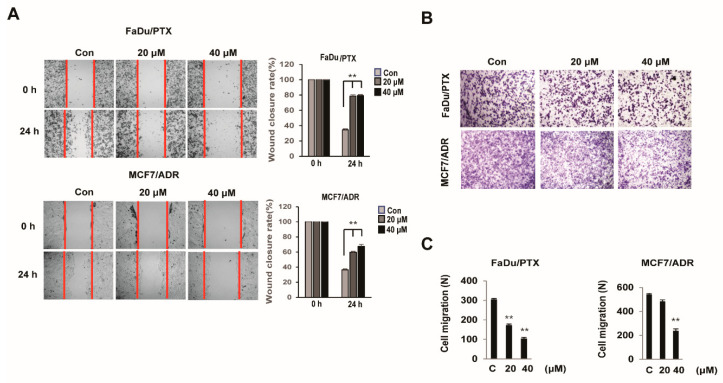
HL156A inhibits cell migration. (**A**) A wound healing assay showed the closure rates of FaDu/PTX and MCF7/ADR cells treated with HL156A (20 or 40 µM) for 24 h. Images of wound-closure rates at the indicated time points. (**B**) The effect of HL156A on cell migration. Cells were treated with HL156A for 22 h. Migration assays were performed as described in the Materials and Methods section. (**C**) The graphs show the quantitative evaluation of the migration rates. The results represent the averages of three independent experiments. ** *p* < 0.01. Data were compared by ANOVA with Bonferroni’s multiple comparisons test, in which each group was compared to the mean of the untreated cells.

To investigate whether HL156A regulates cell migration in MDR cells, a scratch wound healing assay was performed. Treatment with HL156A resulted in a wound closure rate reduction in FaDu/PTX and MCF7/ADR cells in a concentration-dependent manner (Figure 3A). Additionally, HL156A also decreased cell migration, as demonstrated by an in vitro Matrigel–Transwell chamber assay. When FaDu/PTX and MCF7/ADR cells were exposed to 20 or 40 μM HL156A, the cell invasion/migration capability was significantly suppressed. At a concentration of 40 μM, HL156A reduced the number of invaded FaDu/PTX and MCF7/ADR cells to approximately 34% and 43%, respectively, of that in the control cells. (Figure 3B,C).

### 2.4. HL156A Suppresses MDR1 through the HOXC6-ERK1/2 Pathway

A previous study reported that the expression of MDR1 was regulated by the activation/expression of MAPK and HOXC6 [28]. Here, we examined the effects of HL156A on the expression of HOXC6, MDR1 and MAPKs to delineate its mechanism in drug-resistant cells.

**Figure 4:**
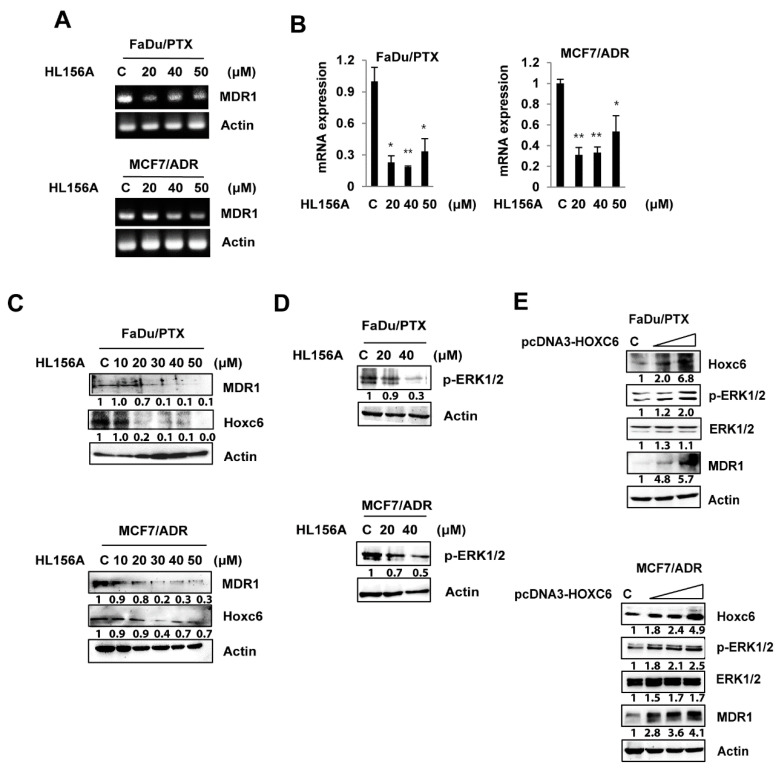
HL156A regulates MDR1 expression through the HOXC6/ERK1/2 pathway. (**A**,**B**) MDR1 mRNA expression was determined by RT-PCR and quantitative real-time PCR analysis. Cells were treated with HL156A (20, 40 and 50 μM) for 24 h. Data are presented as the mean ± SD. * *p* < 0.05 and ** *p* < 0.01. Data were compared by ANOVA with Bonferroni’s multiple comparisons test, in which each group was compared to the mean of the untreated cells. (**C**) MDR1 and HOXC6 expression was measured in FaDu/PTX and MCF7/ADR cells by Western blotting. Cells were treated with HL156A at the indicated concentration for 24 h. (**D**) Western blotting of ERK1/2 phosphorylation after HL156A treatment. (**E**) The effect of HOXC6 on MDR1 expression and ERK1/2 activation. FaDu/PTX and MCF7/ADR cells were transfected with HOXC6 plasmid (pcDNA3-HOXC6) for 48 h. The expression and activation of MDR1, HOXC6, and p-ERK1/2 were detected by Western blotting.

**Figure 5:**
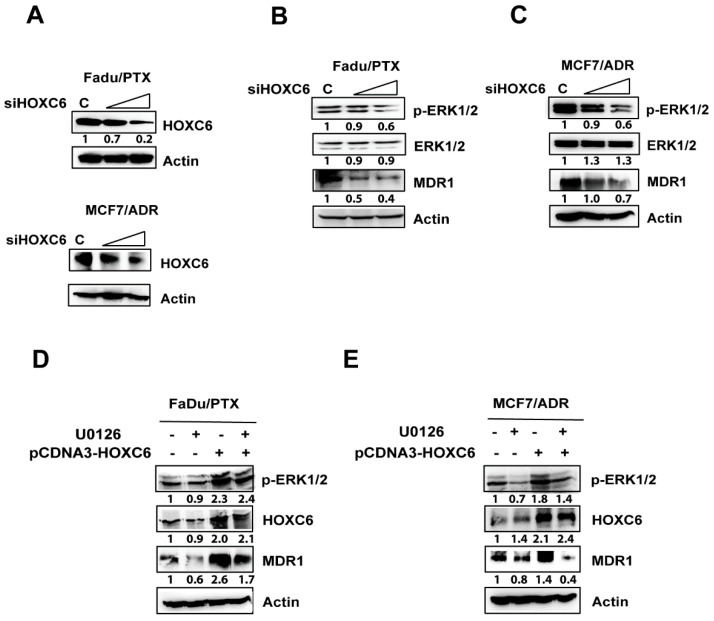
HOXC6 silencing suppresses MDR1 and phospho-ERK1/2 expression. (**A**–**C**) The expression of p-ERK1/2 and MDR1 in FaDu/PTX and MCF7/ADR cells transfected with HOXC6 siRNA. After 48 h of siRNA transfection, p-ERK1/2 and MDR1 expression was determined by Western blot analysis. (**D**,**E**) Effects of the ERK1/2 inhibitor U0126 on HOXC6 and MDR1 expression. HOXC6 transfected cells. were treated with U0126 (50 μM) for 24 h. Whole cell lysates were obtained posttreatment, and the samples were separated by 12% SDS/PAGE and probed for HOXC6, p-ERK1/2, and MDR1, with actin as a loading control.

As shown in Figure 4A, HL156A treatment led to a decrease in MDR1 mRNA expression in both FaDu/PTX and MCF7/ADR cells. In addition, quantitative RT-PCR results also showed that HL156A significantly decreases MDR1 mRNA levels (Figure 5B). Based on qRT-PCR, the expression of MDR1 protein was reduced in both HL156A-treated cells in a concentration-dependent manner (Figure 5C). Consistent with previous research regarding the regulation of MDR1 by HOXC6, HOXC6 and MDR1 expression decreased in a similar manner after HL156A treatment. We also observed that the phosphorylation of ERK1/2 was downregulated by HL156A (Figure 4D). However, JNK and p38 phosphorylation did not change after HL156A treatment (data not shown). These results indicated that HL156A induces the inhibition of MDR1, HOXC6, and pERK1/2.

To identify the correlation between HOXC6 and MDR1 or ERK1/2, we examined the expression of MDR1 or ERK1/2 in HOXC6-overexpressing FaDu/PTX and MCF7/ADR cells. Overexpression of HOXC6 resulted in the upregulation of both MDR1 and p-ERK1/2 (Figure 4E).

### 2.5. siHOXC6 Regulates MDR1 Expression by Suppressing ERK1/2 Activity

To determine the effect of HOXC6 depletion in MDR cells, we performed siRNA silencing of HOXC6. Importantly, HOXC6 silencing led to the reduction in MDR1 and p-ERK1/2 expression in a concentration-dependent manner (Figure 5A–C). In addition, to examine whether ERK inhibition regulates the expression of HOXC6-induced MDR1, we treated HOXC6-overexpressing MDR cells with the ERK-specific inhibitor U0126. The expression of MDR1 via HOXC6 was reduced by U0126 treatment (Figure 5D,E). However, the ERK inhibitor did not affect the expression of HOXC6. These results suggest that HOXC6 regulates MDR1 by activating ERK1/2.

### 2.6. HL156A Enhances Drug Accumulation and Sensitivity in MDR Cells

**Figure 6:**
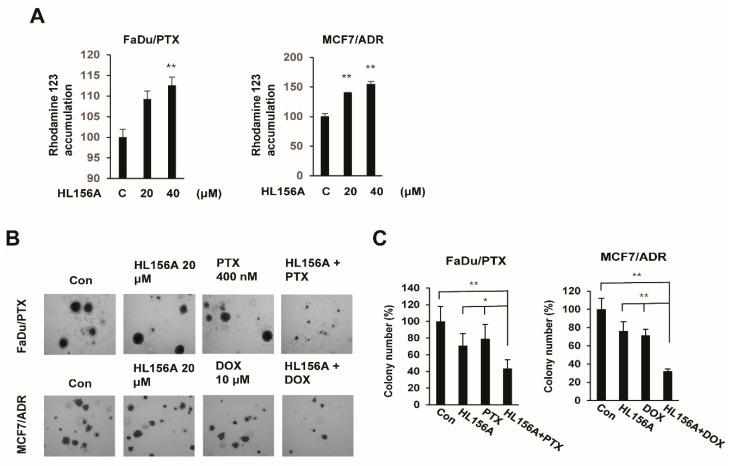
HL156A induces intracellular rhodamine 123 accumulation. (**A**) Representative drug uptake is shown by the levels of Rho123 accumulation in FaDu/PTX and MCF-7/ADR cells treated with 20 µM HL156A for 24 h. Data are presented as the mean ± SD (*n* = 3). ** *p* < 0.01. Data were compared by ANOVA with Bonferroni’s multiple comparisons test. (**B**) Evaluation of the colony formation of FADU/PTX or MCF7/ADR cells cotreated with HL156A (20 µM) and paclitaxel (400 nM) or doxorubicin (10 µM). Colony formation was assessed over 14 days, and cells were stained with crystal violet at the end of the experiment. Images were taken with an inverted microscope at 40× magnification. (**C**) The graphs show the quantitative evaluation of the colony number. Data are presented as the mean ± SD (*n* = 3). * *p* < 0.05, ** *p* < 0.01. Data were compared by ANOVA with Bonferroni’s multiple comparisons test.

To determine whether the inhibition of the HOXC6/ERK1/2/MDR1 pathway by HL156A was sufficient to sensitize the FaDu/PTX and MCF7/ADR cells to chemotherapy, a rhodamine 123 accumulation assay was performed. FaDu/PTX and MCF7/ADR cells were treated with HL156A and administered Rho123. As shown in Figure 6A, Rho123 accumulation significantly increased after treatment with HL156A compared to treatment with the control. At 40 μM, HL156A enhanced rhodamine 123 accumulation by approximately 12% in FaDu/PTX cells and by 50% in MCF7/ADR cells.

The effect of paclitaxel and doxorubicin on the proliferation of FADU/PTX and MCF7/ADR cells was investigated in combination with a low concentration of HL156A (20 μM). Colony formation assays showed that the combination of paclitaxel (400 nM) or doxorubicin (5 μM) and 20 μM HL156A resulted in smaller and fewer colonies than did treatment with each anticancer drug alone (Figure 6B,C).

### 2.7. HL156A Prevents Angiogenesis of Xenografted MDR Cells in the CAM Model

**Figure 7:**
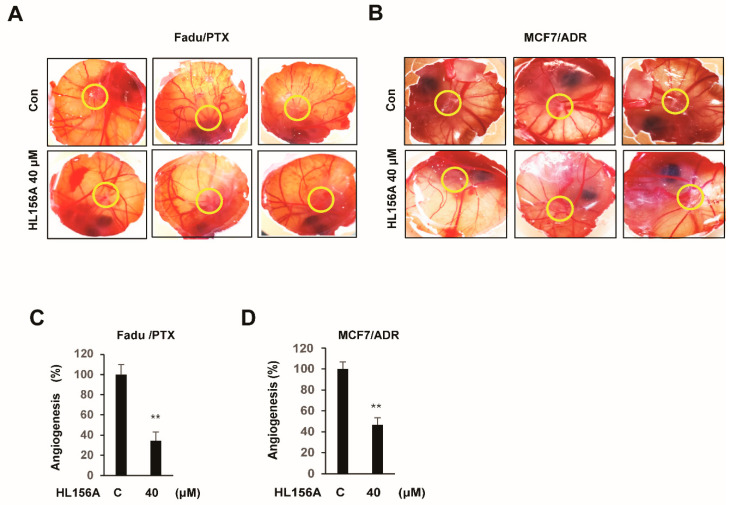
HL156A inhibits angiogenesis in an in vivo CAM model. (**A**,**B**) Fertilized eggs were implanted with HL156A-treated FaDu/PTX or MCF7/ADR cells (1 × 107) for 24 h and incubated in a humidified incubator at 37 °C for an additional 3 days (*n* = 5). (**C**,**D**) The graphs show the quantitative evaluation of angiogenesis. Values are presented as the mean ± SD ** *p* < 0.01. Student’s t-tests were used to determine the significance.

Angiogenesis is considered the most vital process in cancer growth and development. To investigate the effect of HL156A on angiogenesis, an in vivo chick chorioallantoic membrane (CAM) assay was performed. Cells treated with 40 μM HL156A for 24 h were cultured in fertilized eggs for 3 days. It was observed that angiogenesis around the tumor of treated cells was significantly reduced compared to that in the control group (n = 5, Figure 7A,B). The level of angiogenesis (based on the number of counted vessels) was decreased by approximately 70% in FaDu/PTX cells and by 55% in MCF7/ADR cells (Figure 7C,D).

## 3. Discussion

Recently, metformin (1,1-dimethylbiguanide hydrochloride) has attracted great interest by not only reducing the incidence of cancer but also inhibiting the metastasis of various types of cancer [15,29,30]. Additionally, the relationship between metformin and MDR has been reported in breast cancer and hepatocellular carcinoma [31,32]. Metformin exhibits the ability to inhibit the multi drug resistance 1 (MDR1), thus re-sensitizing the cancer cells through the AMPK/mTOR pathway. Treatment with metformin increased the phosphorylation of AMPK and decreased the phosphorylation of mTOR, thus downregulating the MDR1 upstream regulator HIF-1a, and resulting in reduced expression of the MDR1 protein. Additionally, Metformin can also inhibit NF-κB activity and expression and prevent its binding to the MDR-1 promoter, thus reversing the multidrug-resistant phenotype in cancer cells [20,32,33].

MDR1, encoded by P-glycoprotein, has attracted interest because of its role in MDR in a variety of cancers. Its main role is to extrude toxic chemicals out of the cell. P-gp protein recognizes a wide range of chemicals, from natural to artificial ones. In the cancer cells, P-gp expression can be induced by pharmaceutical xenobiotics, such as paclitaxel, rifampicin and tamoxifen, either by the ligand-mediated pathway or through the steroid and xenobiotic receptor SXR [34,35,36,37]. The molecular mechanism of the induction of MDR1 expression has been reported to correlate with cyclooxygenase-2, reactive oxygen species, MAPKs, phosphoinositide 3-kinase and NF-kb [38,39,40,41]. Our previous studies demonstrated that HOXC6 is highly expressed in MDR cells and that HOXC6 induces MDR1 expression [28]. In this study, we investigated the metformin derivative HL156A to identify an effective MDR-reversing agent and to gain insight regarding its molecular mechanism. We found that HL156A strongly inhibits the proliferation of both parental cells and drug-resistant cells (FaDu/PTX and MCF-7/ADR) at lower concentrations (≤100-fold) than metformin. HL156A induced G2/M phase arrest and cell death as well as inhibiting colony formation in FaDu/PTX cells or MCF7/ADR cells. In addition, we also demonstrated that HL156A inhibited the constitutive phosphorylation of ERK1/2 but not JNK or p38 phosphorylation. Interestingly, the phosphorylation of ERK1/2 was induced by the overexpression of HOXC6 and, importantly, was abolished by HOXC6 siRNA. The ERK inhibitor U0126 decreased HOXC6-induced MDR1 expression, suggesting direct regulation of MDR1 through the ERK1/2 pathway.

It has been reported that ERK1/2 regulates the expression of MDR1 in colorectal and breast cancers, and inhibition of the ERK1/2 pathway reduces cell proliferation, metastasis, and angiogenesis [42,43]. Our results suggest that MDR1 inhibition by HL156A is closely associated with inhibition of ERK1/2 signaling through the downregulation of HOXC6. It can partially explain the mechanism of HL156A in drug-resistant cancer cells.

The reduction in MDR1 expression has been proposed to reverse the MDR phenotype [8,10]. We found that HL156A increases the intracellular accumulation of Rho123 in FaDu/PTX cells and MCF7/ADR cells. These results suggested that the accumulation of Rho123 by HL156A inhibits MDR1 expression in these cells. To confirm the anticancer drug sensitivity, FaDu/PTX cells and MCF7/ADR cells were treated with either a nontoxic dose of a chemotherapeutic drug (paclitaxel or doxorubicin), HL156A or both. In the colony formation analysis, the number of colonies was slightly decreased in paclitaxel or doxorubicin-treated cells. Similar to paclitaxel- or doxorubicin-treated cells, HL156A also inhibited colony formation in FaDu/PTX cells and MCF7/ADR cells. Interestingly, treatment with HL156A combined with paclitaxel or doxorubicin significantly inhibited colony formation compared with HL156A or an anticancer drug alone. These results strongly support the hypothesis that HL156A acts on MDR1 activity/expression to mediate drug sensitivity.

Using an in vivo chick chorioallantoic membrane (CAM) model, our results show that HL156A treatment significantly inhibits blood vessel formation in FaDu/PTX and/or MCF7/ADR cell-implanted fertilized eggs. Taken together, these results strongly imply that HL156A inhibits tumor progression not only by inhibiting cell proliferation but also by preventing angiogenesis. However, it is necessary for clinical studies to continually investigate the in vivo effects of HL156A in the future.

In conclusion, our data demonstrate that HL156A suppresses multidrug resistance. First, the drug accumulation of HL156A in FaDu/PTX and/or MCF7/ADR cells strongly indicates that HL156A is attributable to the inhibition of MDR1-mediated efflux. Second, we demonstrated that HL156A suppresses MDR1 expression by inhibiting ERK1/2 phosphorylation through the downregulation of HOXC6. HL156A, a derivative of the antidiabetic drug metformin, could be a new candidate as a potential chemotherapeutic agent in multidrug-resistant cancer cells.

## 4. Materials and Methods

### 4.1. Cell Culture and Reagents

Adriamycin-resistant human breast cancer cells (MCF7/ADR, provided by Dr. Kang KW, Seoul University, Seoul, Korea), paclitaxel-resistant human pharynx squamous cell carcinoma cells (FaDu/PTX) and cisplatin-resistant human gastric cancer cells (SNU601/CIS, provided by Dr. Choi CH, Research Center for Resistant Cells, Chosun University, Gwangju, Korea) were maintained in Dulbecco’s modified Eagle’s medium, minimum essential medium, and RPMI-1640 medium (Welgene Inc., Gyeongsanbuk-do, Gyeongsan-si, Korea), respectively, containing 10% fetal bovine serum (FBS) and 100 units/mL penicillin and 100 μg/mL streptomycin at 37 °C in an incubator with 5% CO_2_/95% air atmosphere. FaDu-PTX cells were created by exposing wild-type FaDu cells to increasing concentrations of paclitaxel for more than 10 months. Adriamycin and paclitaxel were purchased from Sigma Chemical Co. (St. Louis, MO, USA). The ERK1/2 inhibitor U0126 was purchased from Promega Corporation (Madison, WI, USA). HL156A was designed and synthesized by Hanall Biopharma Inc. (Seoul, Korea). Product details are described in a previous study [22].

### 4.2. Cell Proliferation Assay

Cell proliferation was assessed using a 3-(4,5-dimethylthiazol-2-yl)-2,5-diphenyltetrazolium bromide (MTT) assay. The cells were seeded in a 24-well plate at a density of 5 × 104 cells/well. Cells were treated with different concentrations of HL156A ranging from 10 to 50 μM for 24 h or 48 h. Before testing, the MTT solution (5 mg/mL in PBS) was added, and the cells were incubated at 37 °C for 3 h. The culture medium was aspirated, and an acid–isopropanol mixture (0.04 mol/L HCl in isopropanol) was added to dissolve the dark blue crystals. The optical density value of the dissolved solute was measured at a wavelength of 570 nm using an ELISA reader (Beckman Coulter, Inc., Carlsbad, CA, USA). To evaluate DNA synthesis/cell proliferation, the bromodeoxyuridine (BrdU) incorporation assay was performed using Cell Proliferation ELISA kits (1647229; Roche Applied Science, Mannheim, Germany). Briefly, Cells were plated at 5000 cells/well in 96-well culture plates in complete media. After attaining 80% confluence, cells were treated with or without HL156A (10, 30, and 50 µM) for 24 h or 48 h. BrdU solution (10 μM) was added during the last 2 h of stimulation. Next, the cells were dried and fixed, and the cellular DNA was denatured with FixDenat solution for 30 min at room temperature. A peroxidase-conjugated mouse anti-BrdU monoclonal antibody was added to the culture plates and incubated for 90 min at room temperature. Finally, tetramethylbenzidine substrate was added for 5 min at RT and the absorbance of the samples was measured using a microplate reader (DTX880, Beckman Coulter, Inc., CA, USA) at 450 nm.

### 4.3. Soft Agar Colony Formation Assay

In brief, the cells (1 × 104 cells/mL) were suspended in 1 mL of 0.3% basal medium Eagle’s agar containing 10% FBS and were plated in 60-mm plates containing 0.5% low melting temperature agarose. A layer of medium was added to the agar surface to prevent aridity. The cells were allowed to form colonies for 14 days at 37 °C in a humidified atmosphere containing 5% CO_2_. The colonies were fixed with 4% paraformaldehyde for 15 min at room temperature and were then stained with 0.005% crystal violet (Sigma, St. Louis, MO, USA) diluted in 20% methanol for 30 min at room temperature. The cell colonies were scored using an IX2-SLP inverted microscope (Olympus, Tokyo, Japan).

### 4.4. Wound Healing Motility Assay

The cells were plated at a density of 1 × 10^6^ cells in a 6-well plate and allowed to grow in a 5% CO_2_ incubator at 37 °C for 24 h before a scratch ~3-mm wide was created in the cell monolayer using a pipette tip. After being washed twice with PBS, the cells were incubated without or with HL156A. The cells were imaged in 4 random microscopic fields per well using an Olympus IX2-SLP inverted microscope (Olympus, Japan) at ×100 magnification. The distance migrated by the cell monolayer was measured during indicated period, in order to close the wounded area. The distance was determined using ImageJ software.

### 4.5. Cell Migration Assay 

Cell migration was investigated using a two-chamber migration assay with a pore size of 8 mm. In total, 2 × 10^6^ cells were treated with or without HL156A before being resuspended in 200 µL of serum-free medium and seeded on the upper compartment of a 12-well Transwell culture chamber. An amount of 700 µL of complete medium (Welgene, Daegu, Korea) was added to the lower compartment. After incubation at 37 °C, the chambers were washed twice with PBS to remove nonattached cells, fixed in 4% paraformaldehyde solution for 2 min at room temperature and stained with 0.05% crystal violet diluted in 20% methanol for 10 min at room temperature. Images of migrated cells were captured using an Olympus IX2-SLP inverted microscope (Olympus, Japan).

### 4.6. Cell Aycle Analysis and Annexin V-FITC/PI Double Staining

For cell cycle analysis, cells were harvested by trypsinization, washed once in ice-cold 1X PBS, centrifuged at 300× *g*; the resulting cell pellets were fixed in 75% ethanol. Fixed cells were subsequently stained with 250 μL of propidium iodide (PI) solution (20 μg/mL PI, 200 μg/mL DNase-free RNase A in 1X PBS) for 30 min at 37 °C. DNA content was analyzed using a Cell LabQuanta SC flow cytometer (Beckman Coulter Inc., Brea, CA, USA). For apoptosis analysis, cells were stained with the Apoptosis Assay Kit (Invitrogen, Carlsbad, CA, USA), followed by labeling with Alexa Fluor^R^488 annexin V and propidium iodide. For each sample, 10,000 cells were analyzed immediately using the Cell Lab Quanta™ SC flow cytometer (Beckman Coulter, Brea, CA, USA) and software. Apoptotic cells were visualized with an Olympus IX71 fluorescence microscope.

### 4.7. Western Blot Analysis

Harvested cells were incubated with Radioimmunoprecipitation assay buffer (RIPA buffer) (50 mmol/L Tris-Cl (pH 7.5), 150 mmol/L NaCl, 0.5% [*wt*/*vol*] sodium deoxycholate, 1% NP-40 [*vol*/*vol*], 0.1% SDS [*wt*/*vol*] and 1 mmol/L EDTA) containing a protease inhibitor cocktail (1 μg/mL aprotinin and leupeptin) to extract proteins from the cells. Proteins in the cell lysate (30 μg) were separated through SDS-PAGE and transferred to nitrocellulose membranes (Amersham Pharmacia Biotech, Buckinghamshire, UK). Membranes were blocked with 5% skim milk for 3 h before adding primary antibodies against pCDK1 (sc101654), cyclin B1 (sc245), MDR1 (sc55510), and HOXC6 (sc376330), which were purchased from Santa Cruz; PARP (9542S), AIF (5318P), ERK1/2 (9102S), and p-ERK1/2 (9101S) were purchased from Cell Signaling. After being washed twice, the membranes were incubated with the corresponding secondary antibodies (HRP-conjugated-linked anti-mouse IgG or HRP-linked anti-rabbit IgG) for 1 h (dilution ratio 1:5000). Protein signals were analyzed with a luminescent image analyzer (LAS-1000; Fujifilm, Tokyo, Japan) using the Immobilon ™ Western Chemiluminescent HRP Substrate (Millipore, Burlington, MA, USA).

### 4.8. Quantitative RT-PCR

Total RNA was isolated using RNAiso Plus reagent (Takara Bio Inc., Shiga, Japan). Briefly, cells were washed with cold PBS and added to 500 μL of RNAiso Plus reagent/well. Cells were transferred to new tubes, and 100 μL of chloroform was added. After incubation for 5 min at room temperature, samples were centrifuged at 4000 rpm for 10 min. The supernatant was collected, mixed with an equal amount of isopropanol and incubated for 10 min on ice. Total RNA was obtained by dissolving the RNA pellet with RNase-free water after centrifugation.

Quantitative RT-PCR (qRT-PCR) was performed with 2 μg of total RNA from each sample using SuperScript II Reverse Transcriptase (Invitrogen, Carlsbad, CA, USA) based on the manufacturer’s protocol. Primers for qRT-PCR are described in Table 1. The qRT-PCR experiments were performed with SYBR Premix Ex Taq II (Tli RNaseH Plus) (Takara Bio Inc., Shiga, Japan) with detection on a qTOWER3 real-time PCR thermal cycler (Analytik Jena AG, Thuringia, Germany). Triplicates of 20 μL reactions containing primer and cDNA template were used for quantitation. The PCR was carried out as follows: 1 cycle of 95 °C for 10 min followed by 40 cycles of 95 °C for 20 s, 58 °C for 20 s, and 72 °C for 20 s. This was followed by a melt curve beginning at 60 °C and increasing by 1 °C every 6 s, with SYBR green fluorescence measured at every interval. Relative quantitation of the difference between the control and treated samples was performed using qPCRsoft version 3.0 by Analytik Jena AG (Thuringia, Germany). Gene changes were tested for statistical significance (*p* < 0.05) relative to the control by Student’s *t*-test.

### 4.9. siRNA Interference Assay

Briefly, AccuTarget™ Genome-wide predesigned HOXC6 siRNA (Bioneer Corp., Daeheon, Korea) and Lipofectamine 2000 RNAiMAX transfection reagent (Invitrogen, Carlsbad, CA, USA) were separately mixed in 150 μL of Opti-MEM medium (Gibro/Life Technology, Grand lsland, NY, USA). After incubation at room temperature for 5 min, diluted siRNA was added to dilute Lipofectamine^®^ RNAiMAX Reagent (1:1 ratio). The cells were then incubated for 48 h at 37 °C in 5% CO_2_.

### 4.10. Rhodamine Accumulation Assay

The cells were seeded in a 12-well plate and treated with HL156A for 24 h. After washing 2 times with PBS, 12 μM rhodamine 123 (Rho123) was added to each well and incubated for 30 min in a 5% CO_2_ incubator at 37 °C. The cells were washed twice with ice-cold PBS and dissolved in b-butanol. The fluorescence was measured using an ELISA reader (Beckman Coulter, Brea, CA, USA).

### 4.11. Chorioallantoic Membrane (CAM) Assay

Fertilized chicken eggs were purchased, sterilized with 70% ethanol, and incubated for 7 days in an incubator maintained at 37 °C and 90% humidity. After incubating, a small hole ~2 cm in diameter was buffed and drilled gently over the air sac with a nipper not to break the egg shell. The membrane and the CAM were separated carefully to expose the vascular zone, and 1 × 10^7^ cells (treated with or without HL156A) were injected into the section. The eggs were then covered with a 60 mm culture plate using parafilm and incubated for another 3 days in an incubator. At the end of the experimental period, blood vessels were viewed, photographed and quantified by counting the number of blood vessel branch points to examine angiogenesis.

### 4.12. Statistical Analysis

All experiments were performed at least in triplicate. The results are expressed as the mean ± standard deviation (SD). Student’s t-test and one-way analysis of variance (ANOVA) were used to determine the significant difference between the control and experimental groups. *P* values of less than 0.05 were considered statistically significant.

## Figures and Tables

**Table 1:** Primers Sequences.

Gene	Sense	Antisense
HOXC6	CACCGCCTATGATCCAGTGAGGCA	GCTGGAACTGAACACGACATTCTC
MDR1	GACTGTCAGCTGCTGTCTGGGCAA	GCCAAGACCTCTTCAGCTACTGC
GAPDH	AGCCAAAAGGGTCATCATCTCTGC	GCATTGGGATGATCTTGAGGCTG

## Supplementary Materials

The following are available online at https://www.mdpi.com/1424-8247/13/9/218/s1, Figure S1: Effect of HL156A on the proliferation of the FaDu/PTX and MCF7/ADR cells as determined by BrdU assay, Figure S2: Effects of metformin on cell proliferation in human multidrug resistant cancer cells, Figure S3: Effects of HL156A and metformin on cell proliferation in human FaDu, MCF7, and SNU601 cancer cells, Figure S4: Effects of HL156A in SNU601/CIS cells.
