# Supplementary figures and images for: Metformin Derivative HL156A Reverses Multidrug Resistance by Inhibiting HOXC6/ERK1/2 Signaling in Multidrug-Resistant Human Cancer Cells
Source: Pharmaceuticals (Basel). 2020 Aug 28;13(9):218. doi: 10.3390/ph13090218 (PMC7560051; doi:10.3390/ph13090218)

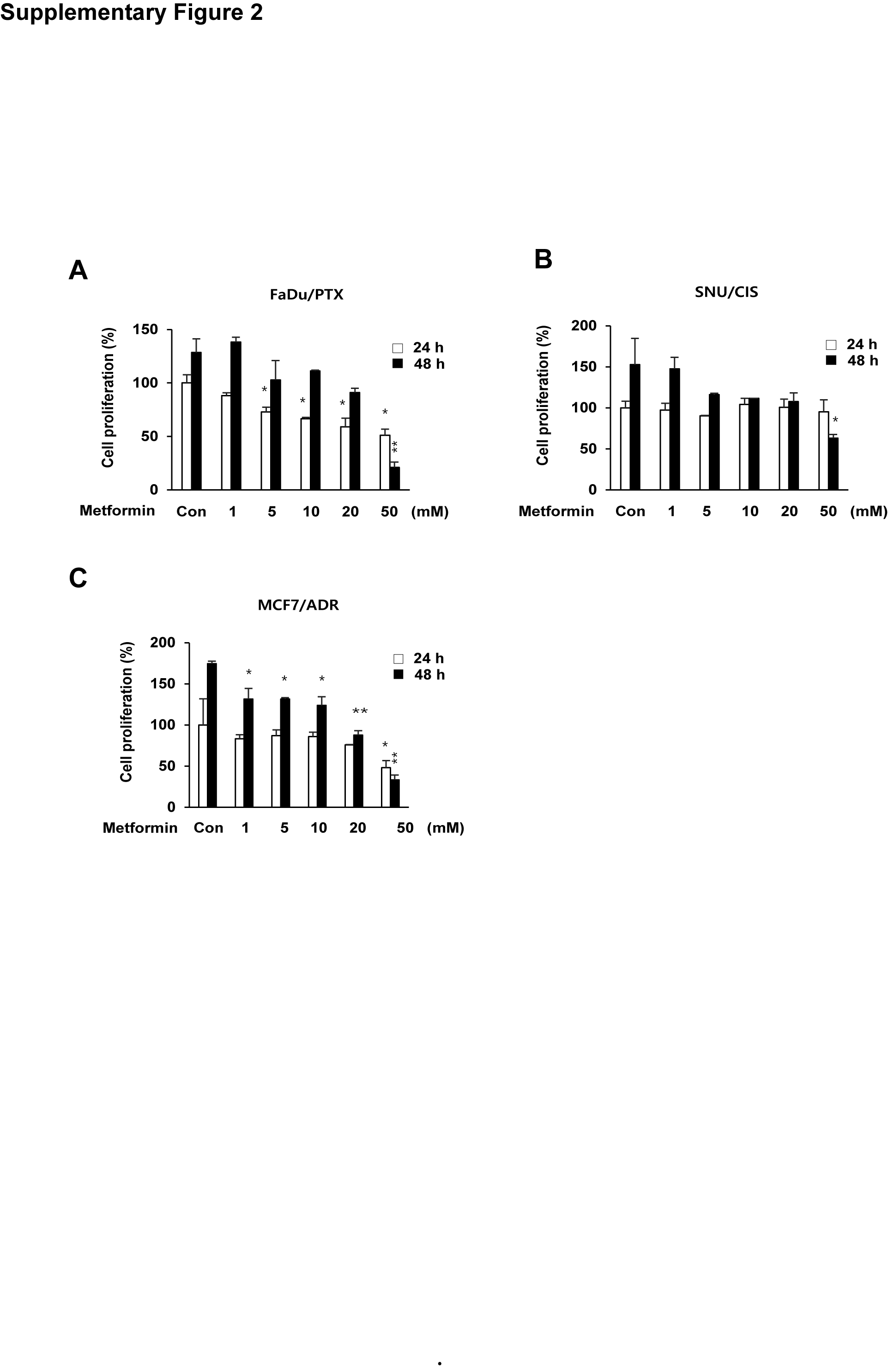

Supplement: Supplementary file 1 [file pharmaceuticals-13-00218-s001.zip › Supplementary Figure 2.tif]

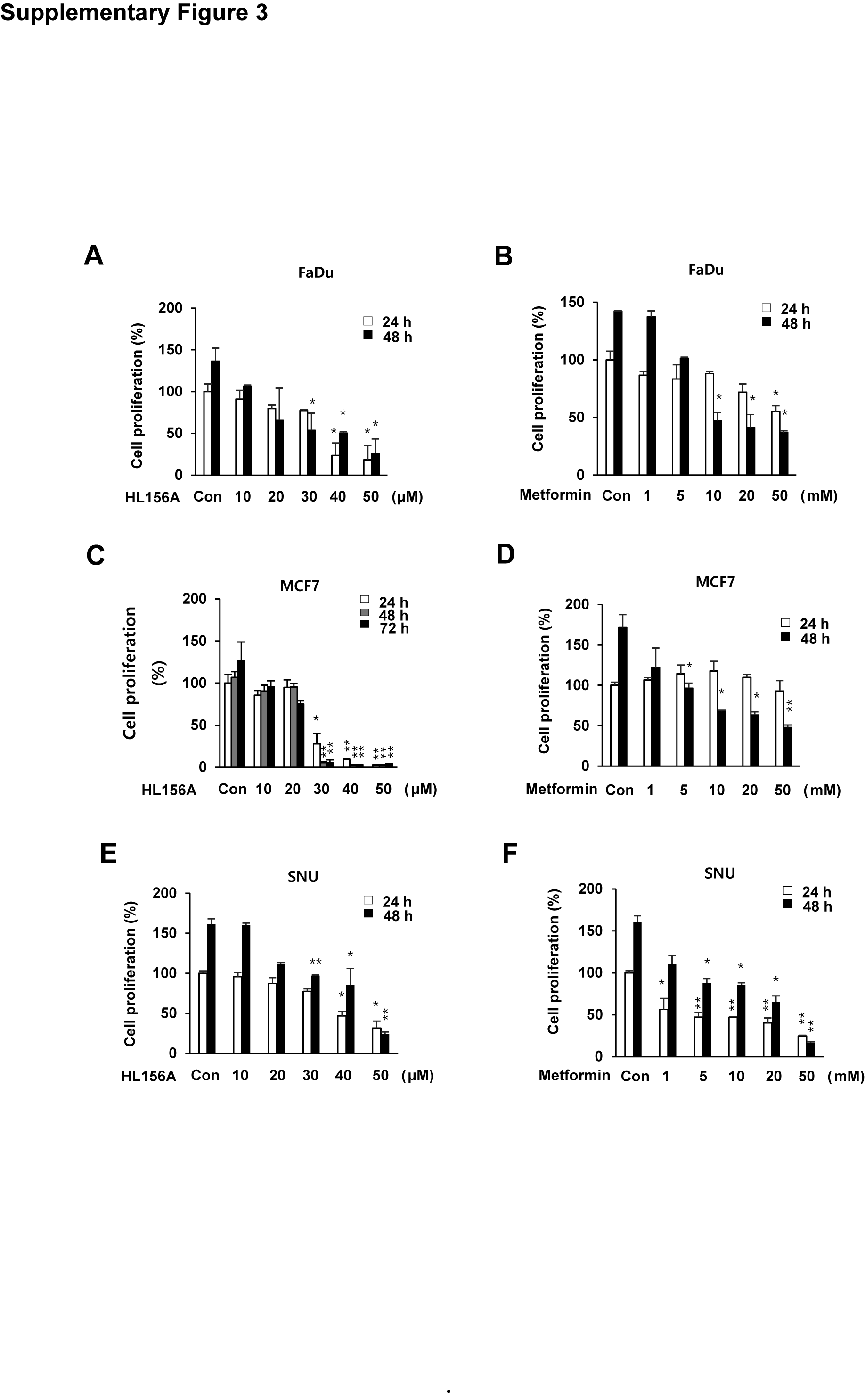

Supplement: Supplementary file 1 [file pharmaceuticals-13-00218-s001.zip › Supplementary Figure 3.tif]

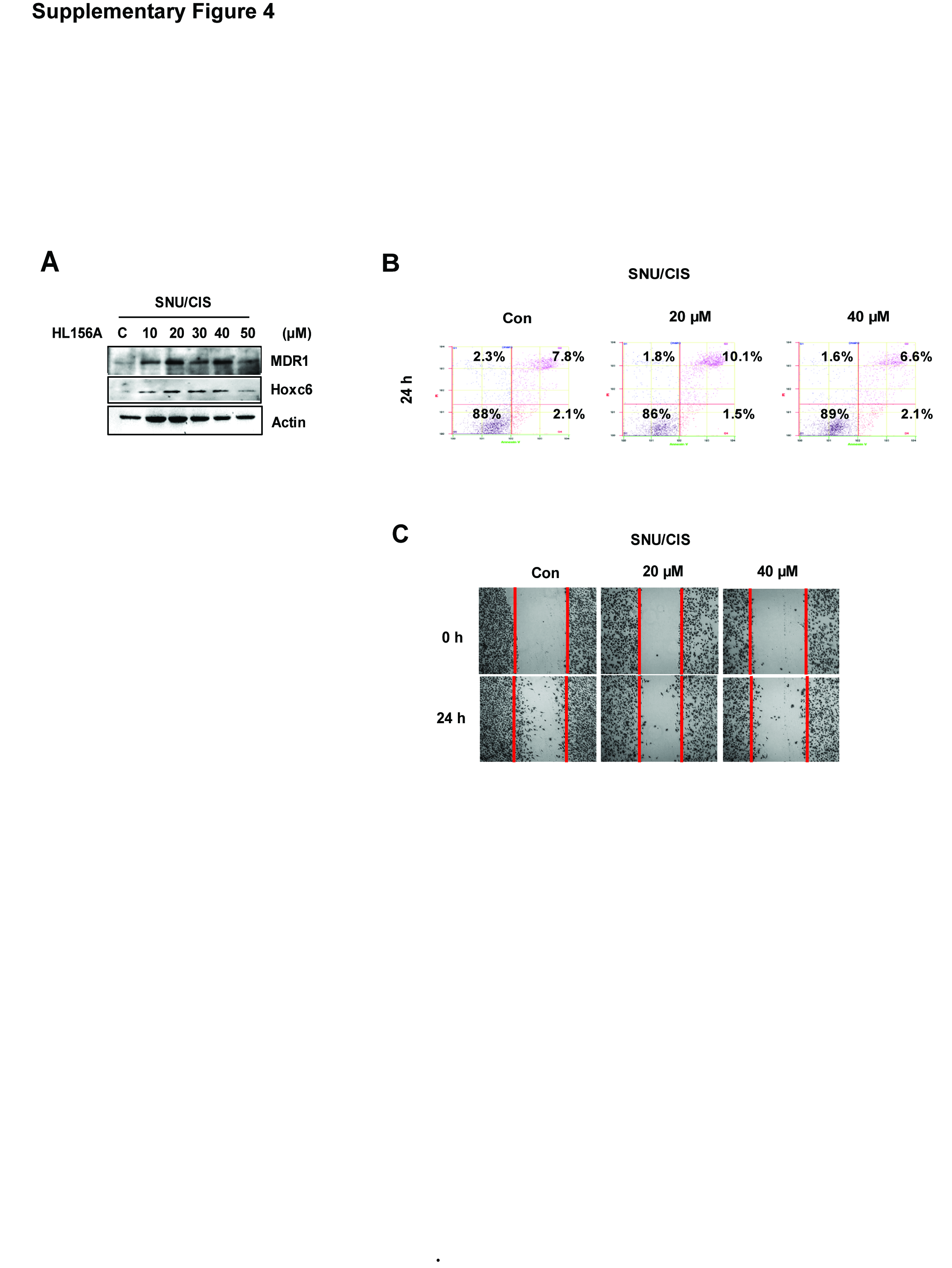

Supplement: Supplementary file 1 [file pharmaceuticals-13-00218-s001.zip › Supplementary Figure 4.tif]

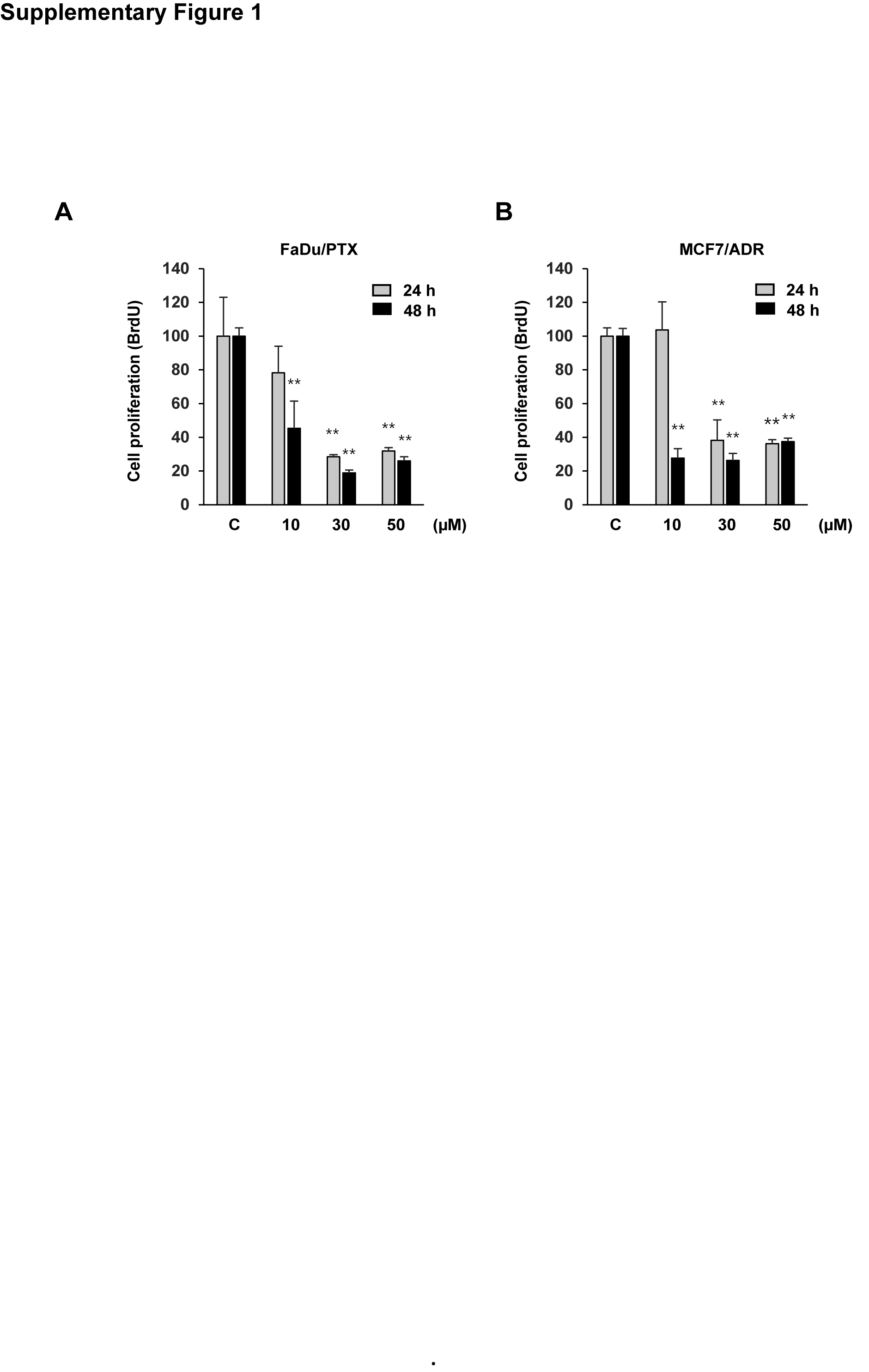

Supplement: Supplementary file 1 [file pharmaceuticals-13-00218-s001.zip › Supplementary Figure 1.tif]
